# Distinct effects of semaglutide and tirzepatide on metabolic and inflammatory gene expression in brown adipose tissue of mice fed a high-fat, high-fructose diet

**DOI:** 10.3389/fnut.2025.1659233

**Published:** 2025-09-11

**Authors:** Tianyi Ma, Fanfan Song, Yongning Pan, Ying He, Xinming Cao, Yan Zhang, Guangyao Song, Luping Ren

**Affiliations:** ^1^Department of Medicine, Hebei Medical University, Shijiazhuang, Hebei, China; ^2^Department of Endocrinology, Hebei General Hospital, Shijiazhuang, Hebei, China; ^3^Hebei Key Laboratory of Metabolic Diseases, Hebei General Hospital, Shijiazhuang, Hebei, China; ^4^Department of Medicine, North China University of Science and Technology, Tangshan, Hebei, China; ^5^Department of Medicine, Hebei North University, Zhangjiakou, Hebei, China

**Keywords:** obesity, high-fat, high-fructose diet, brown adipose tissue, type 2 diabetes mellitus, transcriptional analysis, semaglutide, tirzepatide

## Abstract

**Background:**

Brown adipose tissue (BAT) is crucial for overall energy homeostasis as a thermogenic organ with high metabolic activity. While the recruitment of BAT contributes to improved glycemic and lipid homeostasis, the exact molecular mechanisms remain incompletely understood.

**Objective:**

This investigation compared the transcriptomic responses of semaglutide (GLP-1 receptor agonist) and tirzepatide (dual GIP/GLP-1 receptor agonist) on BAT in mice fed a high-fat, high-fructose diet (HFHFD). These outcomes enhance our understanding of the metabolic actions of GLP-1 and dual GIP/GLP-1 receptor agonists, providing a conceptual basis for future BAT-targeted therapeutic strategies.

**Methods:**

Twenty-eight male C57BL/6J mice were randomly assigned to either a control group (CON; *n* = 7, standard diet) or an obesity model group (*n* = 21, HFHFD). Following the establishment of obesity, the obese mice were further randomized into three intervention groups (*n* = 7) and administered subcutaneous injections of saline, semaglutide, or tirzepatide for 7 weeks. Metabolic parameters (including body weight, glycemic and lipid profiles, and insulin levels) and BAT morphology were assessed. RNA sequencing of BAT was conducted to identify differentially expressed genes (DEGs), followed by gene ontology (GO), Kyoto Encyclopedia of Genes and Genomes (KEGG), and protein–protein interaction (PPI) analyses. Reverse transcription quantitative polymerase chain reaction (RT-qPCR) was subsequently employed to validate the expression of selected DEGs.

**Results:**

Both semaglutide and tirzepatide reduced body weight, improved lipid profiles, and enhanced insulin sensitivity. Compared with the saline group, administration of semaglutide led to differential expression of 467 genes (199 downregulated and 268 upregulated), whereas tirzepatide modulated 40 genes (20 downregulated and 20 upregulated). Bioinformatic analysis identified *Cyp1a1*, *Hsd11b1*, *Atp1a3*, *Tfrc*, *Ptger4*, and *Il1b* as potential therapeutic targets.

**Conclusion:**

Semaglutide and tirzepatide may share common targets (*Cyp1a1*, *Hsd11b1*, and *Atp1a3*) that enhance insulin sensitivity, improve metabolism, and promote weight loss. *Tfrc*, *Ptger4*, and *Il1b* may also serve as tirzepatide-specific targets, potentially elucidating its enhanced anti-inflammatory and metabolic regulatory effects.

## 1 Introduction

As a prototypical lifestyle-related disorder, obesity has escalated into a worldwide epidemic that poses a serious threat to public health and human well-being (1). According to the World Health Organization (WHO), the global prevalence of adult obesity has more than doubled since 1990, while the rate among adolescents has nearly tripled. By 2022, approximately one in every eight individuals worldwide was living with obesity. Individuals with obesity are characterized by a substantially elevated risk for chronic conditions, including type 2 diabetes mellitus and cardiovascular conditions (2). Obesity is typically attributed to a combination of personal factors (such as heredity) and environmental influences, especially unhealthy dietary habits (3).

Incretins refer to a class of gut-derived peptide hormones released upon nutrient ingestion, with glucose-dependent insulinotropic peptide (GIP) and glucagon-like peptide-1 (GLP-1) being the two principal members. GIP receptors (GIPRs) are widely expressed in the pancreas, brain, bone, and adipose tissue (4, 5), whereas GLP-1 receptors (GLP-1Rs) are primarily localized in the pancreas, brain, stomach, liver, and heart (6). Semaglutide, a selective GLP-1 receptor agonist, has received regulatory approval in numerous countries for clinical application in managing type 2 diabetes mellitus (T2DM) and obesity. It can effectively induce weight loss, optimize glucose homeostasis, enhance insulin sensitivity, and provide cardiovascular and renal benefits (7). Tirzepatide is a dual GIP and GLP-1 receptor agonist that has recently gained considerable attention because of its promising clinical efficacy in managing metabolic disease. Iwamoto et al. (8) conducted a comparative study in obese diabetic (db/db) mice treated with either semaglutide or tirzepatide. They found that tirzepatide provided improved glycemic control, better preservation of *β*-cell function, and more substantial reductions in hepatic steatosis and inflammation (8). Similarly, comprehensive analyses of phase 2 (GPWB) and phase 3 (SURPASS 1–5) clinical trials confirmed that tirzepatide outperformed semaglutide in glycemic control, weight reduction, and pancreatic insulin output (9). Despite these advances, the mechanisms underlying the metabolic benefits of GLP-1 receptor agonists and dual GIP/GLP-1 receptor agonists remain poorly understood. In particular, key gaps persist regarding the primary target tissues and potential tissue-biased signaling, the largely unexplored role of BAT, the integration of GIPR- and GLP-1R-mediated downstream pathways, and the mechanisms underlying their anti-inflammatory effects and inter-organ crosstalk.

BAT is functionally distinct from white adipose tissue (WAT), which primarily serves as a lipid storage site. BAT exhibits a specialized form of non-shivering thermogenesis, driven by uncoupling protein 1 (UCP1), which facilitates the direct conversion of chemical energy into heat (10). Studies have shown that cold exposure activates BAT, thereby enhancing plasma triglyceride (TG) clearance (11), improving hypercholesterolemia (12), and increasing glucose uptake (13). It has been reported that, in humans, increased brown adipose tissue (BAT) activity is inversely correlated with body mass index (BMI) (14). However, BAT is more importantly recognized as a metabolically active organ that contributes to whole-body energy homeostasis. There is increasing evidence that stimulation of BAT activity or promotion of WAT browning exerts favorable effects on glucose and lipid metabolism. Although BAT has been implicated in energy homeostasis, its precise role and therapeutic potential remain inadequately supported by conclusive mechanistic evidence. Previous studies have shown that semaglutide enhances the BAT metabolic activity (15) and promotes WAT browning, thereby increasing overall energy expenditure (16). Moreover, Samms et al. (17) demonstrated that tirzepatide improves systemic insulin sensitivity independently of body weight reduction. By utilizing a high-fat diet-induced obese mouse model, they applied pharmacological interventions, hyperinsulinemic-euglycemic clamp studies, as well as transcriptomic and metabolomic analyses to demonstrate that this effect was associated with the activation of glucose, lipid, and branched-chain amino acid (BCAA) metabolic pathways in BAT (17). However, head-to-head comparisons of semaglutide and tirzepatide regarding their transcriptomic regulatory effects on BAT remain insufficiently explored. Whether BAT contributes significantly to the metabolic benefits of incretin-based therapies remains unclear.

Therefore, this study investigated and compared transcriptomic changes in BAT following semaglutide and tirzepatide administration in HFHFD-fed mice, aiming to elucidate their transcriptional regulatory mechanisms and assess whether BAT represents a novel therapeutic target for metabolic disorders. These findings may contribute to a better understanding of the metabolic regulatory mechanisms of GLP-1 and dual GIP/GLP-1 receptor agonists while providing a theoretical basis for future BAT-targeted therapies.

## 2 Materials and methods

### 2.1 Reagents and animals

Tirzepatide (Eli Lilly and Company, Indianapolis, USA) and semaglutide (Novo Nordisk A/S, Bagsværd, Denmark) were purchased from their respective manufacturers. Twenty-eight male C57BL/6J mice (7 weeks old; 20 ± 2 g) were maintained under standardized conditions (24 ± 1 °C, 12-h light/dark cycle) and allowed unrestricted access to regular chow and water. The study protocol was evaluated and endorsed by the Ethics Committee of Hebei General Hospital (Approval No. 2025-DW-002).

### 2.2 Experimental design

Mice were acclimated for one week prior to randomization into either an HFHFD-induced obesity model group (*n* = 21) or a control group (*n* = 7). Mice in the model group received an HFHFD (M18101801: 40% fat, 34.5% fructose, 20% protein, and 2% cholesterol; Biopike Biotechnology, Sichuan, China), whereas those in the control group were fed a standard chow diet (D12450B: 10% fat, 20% protein, and 70% carbohydrates; Research Diets, Inc., New Brunswick, USA). The dietary intervention lasted for 7 weeks, during which body weight was measured weekly. Notably, the HFHFD used in this study contained a higher proportion of fructose compared with conventional diets used in previous models, which was optimized based on our preliminary experiments. This modification was designed to induce more pronounced disturbances in glucose and lipid metabolism, thereby facilitating the establishment of a stable and reproducible metabolic disorder model. At the end of this 7-week feeding period, mice were fasted for 8 h and subsequently subjected to an intraperitoneal glucose tolerance test (IPGTT). Mice received an intraperitoneal injection of 50% glucose solution at a dose of 2 g/kg body weight. Blood glucose concentrations were measured at 0, 15, 30, 60, and 120 min post-injection from tail vein blood using an ACCU-CHEK Performa glucometer (Roche Diagnostics GmbH, Mannheim, Germany). The area under the curve (AUC) was calculated to assess glucose tolerance.

**Figure 1:**
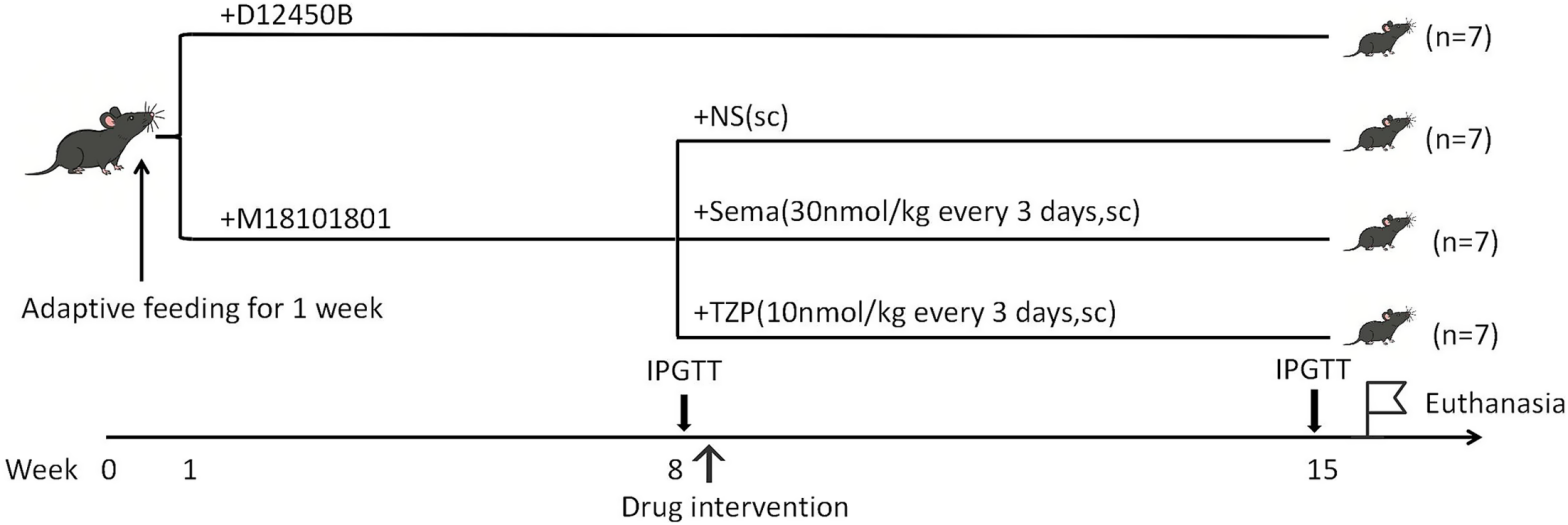
Experimental design and intervention schedule.

Following obesity induction, the model mice (*n* = 21) were arbitrarily distributed across three intervention arms (*n* = 7 per group): semaglutide, tirzepatide, and saline control. Each group received subcutaneous injections of semaglutide (30 nmol/kg), tirzepatide (10 nmol/kg), or an equivalent volume of saline once every 3 days over a 7-week period, with the timeline illustrated in Figure 1. The selected doses were based on established preclinical studies in diet-induced obese C57BL6 mice (17–20), as supported by the cited literature. Specifically, semaglutide at 30 nmol/kg has been widely employed to achieve sustained weight loss and glycemic improvement (18, 21), while tirzepatide at 10 nmol/kg has been validated to produce comparable or greater metabolic benefits due to its higher potency and dual GIP/GLP-1 receptor agonism (17, 20). Both regimens fall within the pharmacologically effective range reported in the literature and were administered every 3 days to maintain therapeutic exposure, as described in previous studies (18, 21).

Body weight was tracked weekly across the intervention timeline. After completing all 16 drug administrations, mice were subjected to a second IPGTT following the same protocol as the first test to evaluate glucose metabolism, and the AUC was calculated accordingly. Mice were intraperitoneally anesthetized with 1% pentobarbital sodium (60 mg/kg; Sinopharm Chemical Reagent Co., Ltd., Shanghai, China) and euthanized via cervical dislocation at the end of the experiment. Blood samples were collected via retro-orbital bleeding, and both interscapular BAT and liver tissues were harvested for subsequent analyses.

### 2.3 Biochemical assays

Blood samples obtained as described in Section 2.2 were centrifuged (3,000 rpm, 15 min, 4 °C), and the serum was stored at −80 °C until analysis. Serum insulin levels were measured using a Mouse Ultrasensitive Insulin ELISA kit (ALPCO, Salem, USA; 80-INSMSU-E01). Serum TG, total cholesterol (TC), high-density lipoprotein cholesterol (HDL-C), and low-density lipoprotein cholesterol (LDL-C) were quantified using commercial assay kits (Nanjing Jiancheng Bioengineering Institute, Nanjing, China; A110-1-1, A111-1-1, A112-1-1, A113-1-1).

For hepatic TG measurement, approximately 50 mg of liver tissue was homogenized with nine volumes of buffer (w/v = 1:9, g: mL) on ice using a mechanical homogenizer. The homogenate was centrifuged (2,500 rpm, 10 min, 4 °C), and the supernatant was used for TG quantification with a commercial kit (Nanjing Jiancheng Bioengineering Institute, Nanjing, China; A110-1-1). Absorbance was measured at 500 nm on a microplate reader (Multiskan FC, Thermo Fisher Scientific, Shanghai, China), and hepatic TG levels were normalized to tissue weight and expressed as mmol/g tissue.

### 2.4 Histological analysis

For hematoxylin and eosin (H&E) staining, BAT tissues were immersed in 10% neutral-buffered formalin for fixation, followed by sequential dehydration in graded ethanol, xylene clearing, and subsequent paraffin embedding. Paraffin-embedded BAT tissues were sectioned into 5-μm slices, deparaffinized, rehydrated, and stained with H&E following standard protocols. After dehydration and cover slipping, tissue morphology was examined under a light microscope at 200 × magnification. For Oil Red O (ORO) staining, BAT samples were rapidly frozen, cryo-sectioned, and treated with 4% paraformaldehyde for fixation. Tissue slices were stained with ORO to visualize lipid droplets, followed by differentiation, hematoxylin counterstaining, rinsing, and mounting. The lipid droplet area was quantitatively analyzed based on microscopic images using Aipathwell software (Servicebio, Wuhan, China).

### 2.5 Transcriptomic analysis

#### 2.5.1 RNA extraction and library preparation

BAT RNA was extracted with TRIzol reagent (Invitrogen, Carlsbad, USA). The assessment of RNA quantity and purity was carried out on a NanoDrop 2000 spectrophotometer (Thermo Fisher Scientific, Wilmington, USA), while RNA integrity was evaluated with an Agilent 2100 Bioanalyzer (Agilent Technologies, Santa Clara, CA, USA). Library construction for RNA sequencing was completed with the VAHTS Universal V5 RNA Library Prep Kit (Vazyme Biotech Co., Ltd., Nanjing, China).

#### 2.5.2 Transcriptomic data processing and bioinformatics analysis

Sequencing was performed using the Illumina NovaSeq 6000 platform (Illumina, San Diego, CA, USA), yielding 150 bp paired-end reads. Adapter trimming and quality filtering of raw FASTQ reads were conducted with fastp (v0.20.1; HaploX Biotechnology, Shenzhen, China) (22), resulting in high-quality clean reads. Clean reads were aligned against the GRCm38/mm10 mouse reference genome via HISAT2 (v2.1.0; Johns Hopkins University, Baltimore, USA) (23). Gene expression was quantified with FPKM (21), and HTSeq-count (v0.11.2; European Molecular Biology Laboratory, Heidelberg, Germany) was applied to generate gene-level read counts (24).

Principal component analysis (PCA) was performed in R (v3.2.0; R Foundation for Statistical Computing, Vienna, Austria) to assess intergroup consistency and clustering patterns. Genes exhibiting a q-value below 0.05 and an absolute log₂ fold change greater than 1 were categorized as differentially expressed based on DESeq2 (v1.22.2; European Molecular Biology Laboratory, Heidelberg, Germany) analysis (25). DEGs were visualized by volcano plots to highlight significantly upregulated and downregulated genes. Hierarchical clustering and heatmap analysis illustrated expression patterns across the groups. Venn diagrams were generated to identify overlapping DEGs among comparison groups.

GO (26) and KEGG (27) enrichment analyses were performed using clusterProfiler (v4.6.0; The University of Hong Kong, Hong Kong, China) in R, with significance determined by hypergeometric testing (*p* < 0.05). Bubble plots depicting gene ratios and significance levels were generated in R. PPI analysis of the top 30 DEGs was conducted with the STRING database (v11.0; Swiss Institute of Bioinformatics, Lausanne, Switzerland, and European Bioinformatics Institute, Hinxton, UK), and the resulting interaction networks were constructed to identify hub genes involved in key regulatory pathways.

### 2.6 RT-qPCR validation procedure for DEGs

To validate the RNA-seq–identified genes associated with inflammation and lipid metabolism, RT-qPCR analysis was performed. Total RNA (0.5 μg) was reverse-transcribed into cDNA using the TransScript All-in-One First-Strand cDNA Synthesis SuperMix with gDNA Remover (TransGen Biotech, Beijing, China; AT341-02). The reverse transcription step was conducted at 42 °C for 15 min, followed by enzyme inactivation at 85 °C for 5 s. Quantitative PCR was carried out using PerfectStart™ Green qPCR SuperMix (TransGen Biotech, Beijing, China; AQ601) on a Roche LightCycler 480 II system (Roche Diagnostics, Basel, Switzerland). Each 10 μL reaction consisted of diluted cDNA, 2 × SuperMix, gene-specific primers (OE Biotech Co., Ltd., Shanghai, China), and nuclease-free water. The primer sequences for the target genes are provided in Supplementary Table S1. The thermal cycling protocol included an initial denaturation at 94 °C for 30 s, followed by 45 cycles of 94 °C for 5 s and 60 °C for 30 s. A melting curve analysis was performed from 60 °C to 97 °C. Relative gene expression was normalized to GAPDH and calculated using the 2^−ΔΔCt^ method.

### 2.7 Statistical analysis

Statistical analyses were carried out using SPSS (version 27.0; IBM Corp., Armonk, USA) and GraphPad Prism (version 9.5; GraphPad Software, San Diego, USA). Results are shown as mean ± standard deviation (SD). Statistical comparisons were carried out using an independent samples t-test, one-way analysis of variance (ANOVA), or two-way ANOVA, based on data distribution and group structure. A threshold of *p* < 0.05 was adopted to indicate statistical significance.

## 3 Results

### 3.1 HFHFD induces significant weight gain and glucose intolerance in mice

**Figure 2:**
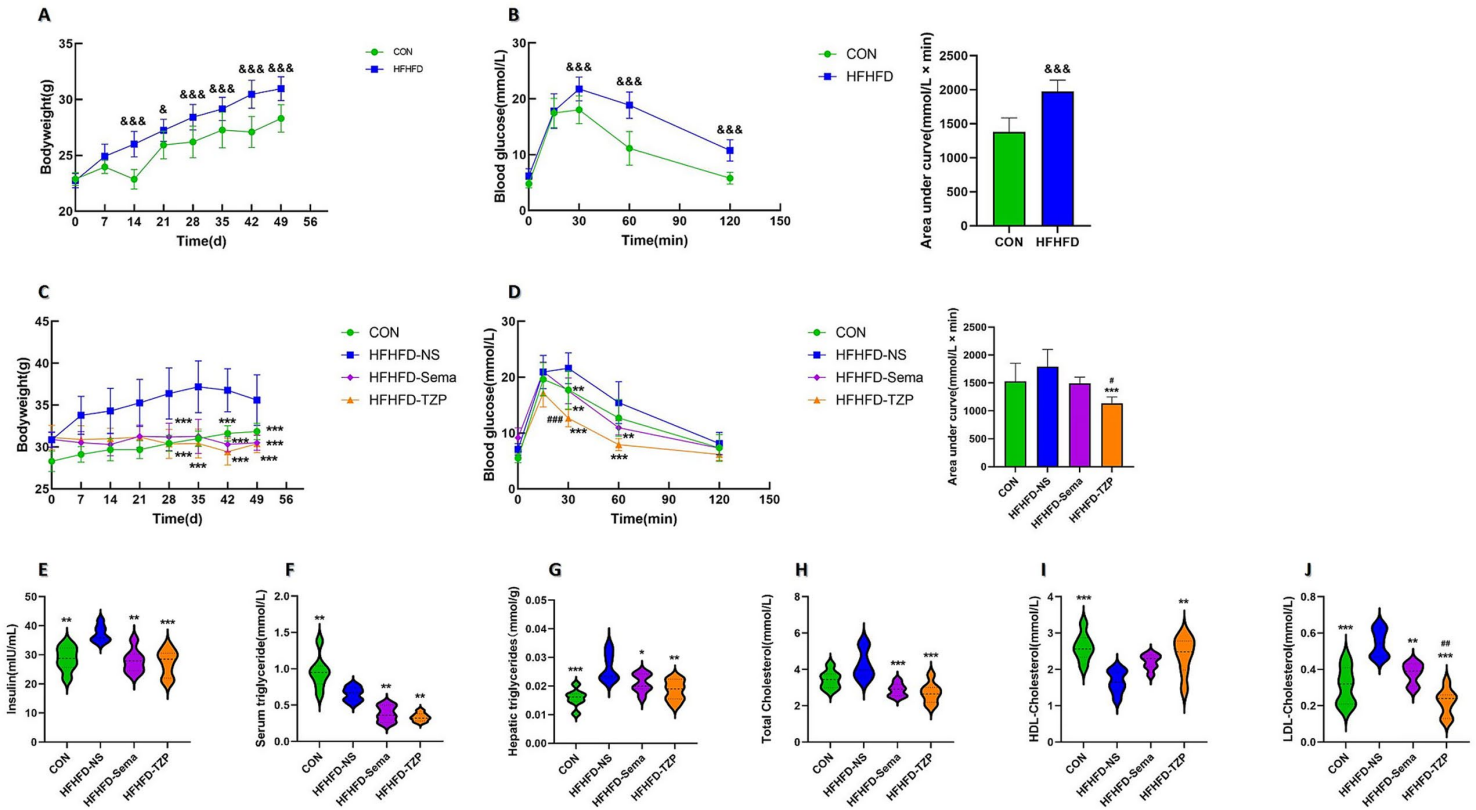
Changes in body weight, blood glucose, lipid profiles, and insulin levels in mice. **(A)** Body weight changes in CON and HFHFD groups after 7 weeks of feeding. Statistical analysis was performed using two-way ANOVA followed by Tukey’s *post-hoc* test. **(B)** Blood glucose levels and AUCs during IPGTT in CON and HFHFD groups. Statistical analysis was performed using two-way ANOVA (for glucose curves) or independent samples t-test (for AUCs), with Tukey’s *post-hoc* test applied after ANOVA. **(C)** Body weight changes in the four groups (CON, HFHFD-NS, HFHFD-Sema, HFHFD-TZP) during intervention. Statistical analysis was performed using two-way ANOVA followed by Tukey’s *post-hoc* test. **(D)** Blood glucose levels and AUCs during IPGTT in the four groups. Statistical analysis was performed using two-way ANOVA (for glucose curves) or one-way ANOVA (for AUCs), followed by Tukey’s *post-hoc* test. **(E–J)** Serum insulin, serum TG, hepatic TG, serum TC, serum HDL-C, and serum LDL-C levels after intervention. Statistical analysis was performed using one-way ANOVA followed by Tukey’s *post-hoc* test. Symbols: &*p* < 0.05, &&*p* < 0.01, &&&*p* < 0.001 vs. CON; *p < 0.05, **p < 0.01, ***p < 0.001 vs. HFHFD-NS; #*p* < 0.05, ##*p* < 0.01, ###*p* < 0.001 vs. HFHFD-Sema.

After 7 weeks of dietary intervention, animals receiving the HFHFD exhibited significantly higher body weight (30.97 ± 1.07 g) compared to the standard diet-fed controls (28.30 ± 1.20 g; *p* < 0.001) (Figure 2A). Following intraperitoneal glucose administration, blood glucose concentrations at 30, 60, and 120 min were significantly elevated in HFHFD-fed mice relative to controls (*p* < 0.001) (Figure 2B). Area under the curve (AUC) analysis further revealed impaired glucose clearance in the HFHFD group (1,975 ± 165.9 vs. 1,379 ± 207.1 mmol/L × min; *p* < 0.001), confirming the development of glucose intolerance.

### 3.2 Effects of semaglutide and tirzepatide on body weight and glucose homeostasis

Following semaglutide or tirzepatide administration, mice exhibited significantly lower body weight compared to the saline group (saline: 35.61 ± 3.03 g; semaglutide: 30.50 ± 0.89 g; tirzepatide: 30.39 ± 1.06 g; *p* < 0.001), representing reductions of approximately 14.35 and 14.66%, respectively (Figure 2C). Following glucose loading, blood glucose levels at 30 and 60 min were significantly reduced in the semaglutide (*p* < 0.01) and tirzepatide groups (*p* < 0.001) compared with the saline group (Figure 2D). Notably, at the 30-min time point, mice administered with tirzepatide exhibited significantly lower blood glucose concentrations (12.67 ± 1.53 mmol/L) than those receiving semaglutide (17.57 ± 2.31 mmol/L; *p* < 0.001). AUC analysis indicated impaired glucose clearance among saline-treated mice relative to all other intervention groups. Furthermore, tirzepatide administration resulted in a lower AUC value (1,132 ± 115.7 mmol/L × min) than semaglutide (1,492 ± 113.2 mmol/L × min), though the difference did not reach statistical significance.

### 3.3 Effects of pharmacological intervention on lipid metabolism and insulin sensitivity

As shown in Figure 2E, the saline group exhibited significantly higher serum insulin concentrations (37.21 ± 2.83 mIU/mL) than the control (28.65 ± 4.18 mIU/mL; *p* < 0.01), semaglutide (27.87 ± 4.03 mIU/mL; *p* < 0.01), and tirzepatide groups (27.2 ± 4.59 mIU/mL; *p* < 0.001), suggesting improved insulin sensitivity following pharmacological intervention. Similarly, serum TG concentrations in the semaglutide (0.38 ± 0.11 mmol/L; *p* < 0.01) and tirzepatide (0.33 ± 0.06 mmol/L; *p* < 0.01) groups exhibited a significant reduction relative to those in the saline-treated group (0.65 ± 0.10 mmol/L) (Figure 2F). When compared to the saline-treated group (0.026 ± 0.0049 mmol/g liver), a significant reduction in hepatic TG accumulation was noted in the control (0.015 ± 0.0031 mmol/g liver; *p* < 0.001), semaglutide (0.021 ± 0.0033 mmol/g liver; *p* < 0.05), and tirzepatide (0.018 ± 0.0036 mmol/g liver; *p* < 0.01) groups (Figure 2G). Unexpectedly, serum TG concentrations in the saline group were lower than those in control animals (0.95 ± 0.23 mmol/L; *p* < 0.01). Comparable findings have been reported in earlier research. In these studies, C57BL/6 J mice fed a choline-deficient L-amino acid-defined high-fat diet (CDAHFD) or a conventional high-fat diet (HFD) exhibited reduced serum TG levels compared with mice on a standard chow diet, despite increased hepatic TG accumulation and more severe hepatic steatosis. This paradoxical reduction in serum TG, concurrent with hepatic TG accumulation, suggests a state of metabolic imbalance. This effect may be attributable to the downregulation of microsomal triglyceride transfer protein (MTP), a critical regulator of very low-density lipoprotein (VLDL) assembly and hepatic TG export (28, 29).

Figure 2H illustrates that TC concentrations were significantly higher in the saline group (4.28 ± 0.82 mmol/L) compared to the semaglutide (2.86 ± 0.36 mmol/L; *p* < 0.001) and tirzepatide (2.72 ± 0.57 mmol/L; *p* < 0.001) groups. Figure 2I shows that HDL-C levels were significantly decreased in the control (2.61 ± 0.38 mmol/L; *p* < 0.001) and tirzepatide (2.39 ± 0.52 mmol/L; *p* < 0.01) groups compared with the saline group (1.66 ± 0.31 mmol/L). In contrast, HDL-C levels in the semaglutide group (2.20 ± 0.21 mmol/L) exhibited a non-significant upward trend (*p* > 0.05). As illustrated in Figure 2J, LDL-C levels were significantly elevated in the saline group (0.54 ± 0.08 mmol/L) relative to the control (0.31 ± 0.10 mmol/L; *p* < 0.001), semaglutide (0.38 ± 0.06 mmol/L; *p* < 0.01), and tirzepatide (0.21 ± 0.07 mmol/L; *p* < 0.001) groups. Notably, tirzepatide reduced LDL-C levels more effectively than semaglutide (*p* < 0.01). These results demonstrate that both semaglutide and tirzepatide ameliorate lipid metabolic abnormalities in HFHFD-fed mice, with tirzepatide exerting more pronounced lipid-lowering effects than semaglutide.

### 3.4 Histological assessment of BAT following pharmacological intervention

**Figure 3:**
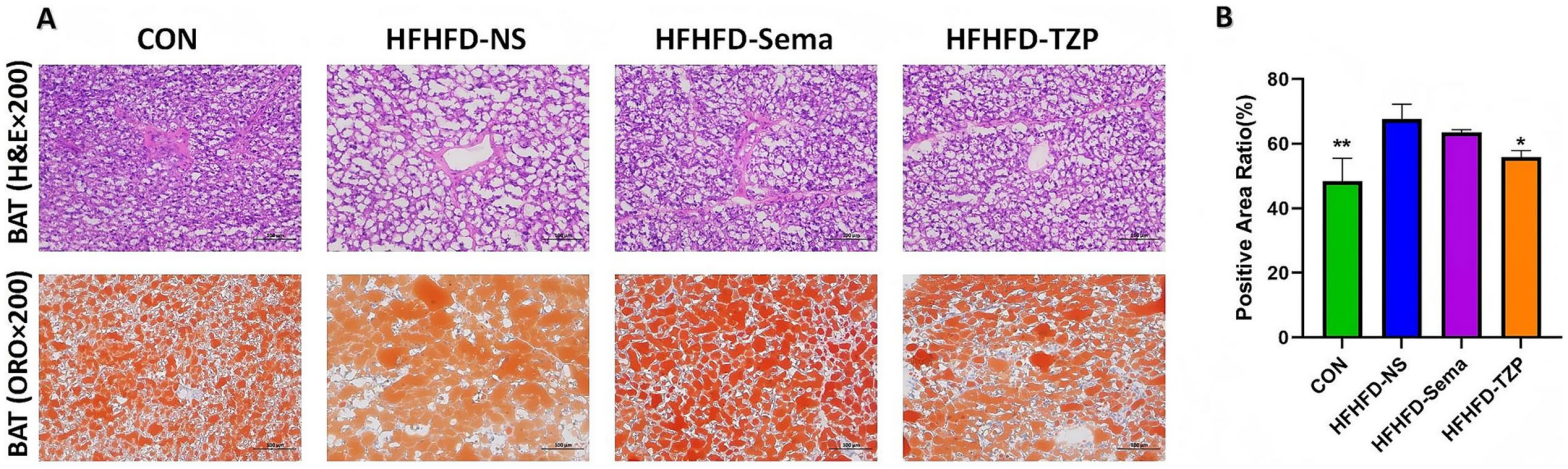
Histological evaluation of BAT in mice following intervention. **(A)** Representative H&E and ORO staining of BAT. H&E staining illustrates lipid droplet morphology, while ORO staining highlights intracellular lipid accumulation (red). Images were captured at 200× magnification; scale bars = 100 μm. **(B)** Quantification of the ORO-positive area ratio. Statistical analysis was performed using one-way ANOVA followed by Tukey’s *post-hoc* test. Symbols: *p < 0.05, **p < 0.01, ***p < 0.001 vs. HFHFD-NS.

H&E staining demonstrated that lipid droplets in BAT presented as round or oval vacuoles of heterogeneous sizes, as observed under light microscopy (Figure 3A). Compared to the saline group, the control, semaglutide, and tirzepatide groups exhibited smaller lipid droplets, suggesting reduced lipid accumulation in BAT. ORO staining revealed lipid droplets uniformly stained red within the cytoplasm, indicating intracellular lipid accumulation. The control, semaglutide, and tirzepatide treatments decreased lipid droplet size and reduced ORO-positive staining areas relative to saline administration. Tirzepatide (55.81 ± 2.09%; *p* < 0.05) and control treatment (48.45 ± 7.07%; *p* < 0.01) significantly reduced the ORO-positive area ratio compared to the saline group (67.68 ± 4.57%) (Figure 3B). Although the semaglutide group (63.56 ± 0.81%) also showed a downward trend relative to the saline group, this difference was not statistically significant (*p* > 0.05). These findings suggest that semaglutide and tirzepatide may ameliorate lipid metabolic abnormalities in BAT.

### 3.5 Transcriptomic profiling and identification of DEGs

**Figure 4:**
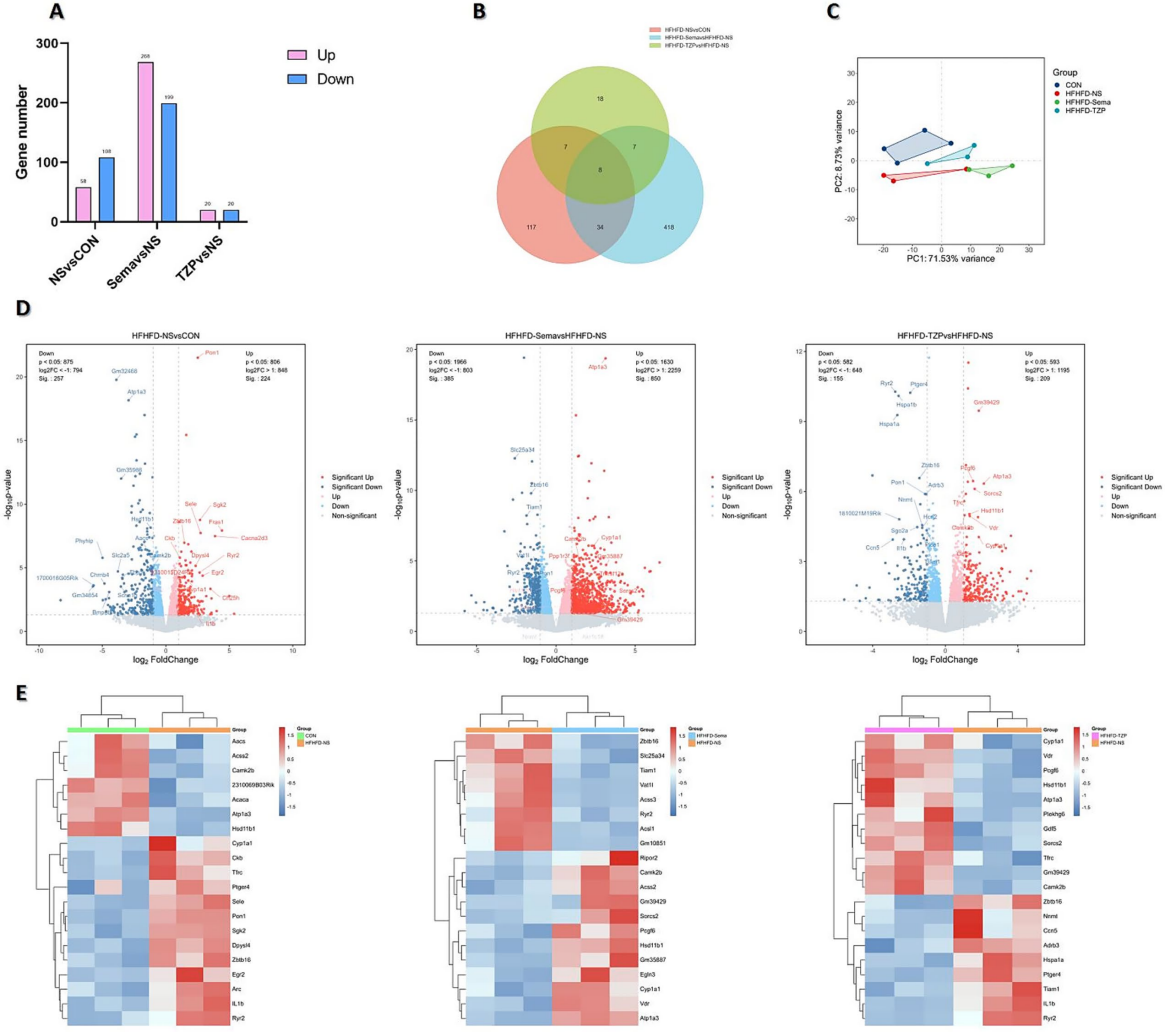
Transcriptomic analysis of DEGs across four groups of mice. **(A)** Number of DEGs identified in each pairwise comparison. **(B)** Venn diagram illustrating the overlap and group-specific DEGs. **(C)** PCA showing distinct clustering and separation of transcriptomic profiles. **(D)** Volcano plots depicting the distribution and magnitude of gene expression changes. **(E)** Heatmaps displaying the expression patterns of DEGs.

RNA-Seq was conducted to assess mRNA expression profiles in BAT and to investigate transcriptional alterations across the four groups. Analysis revealed 58 genes with increased and 108 with decreased expression in the saline group as opposed to the control. Compared with the saline group, semaglutide administration resulted in 467 DEGs, including 268 upregulated and 199 downregulated. In contrast, tirzepatide administration yielded 40 DEGs (20 upregulated and 20 downregulated) (Figure 4A).

Venn diagram analysis (Figure 4B) identified eight DEGs common to all three pairwise comparisons. These genes may participate in shared regulatory pathways related to HFHFD-induced metabolic dysfunction and the therapeutic actions of semaglutide and tirzepatide. Moreover, semaglutide and tirzepatide interventions resulted in 418 and 18 unique DEGs, respectively, underscoring distinct transcriptional responses and possibly different underlying mechanisms. Altogether, 34 DEGs overlapped across the semaglutide and tirzepatide groups, indicating that the two agents may share specific regulatory pathways or therapeutic targets. These results underscore the intricate relationship between HFHFD-driven pathophysiology and drug-mediated transcriptomic modulation. While shared DEGs may serve as core therapeutic targets, drug-specific DEGs likely represent molecular signatures underlying the unique regulatory profiles of each agent.

PCA demonstrated distinct transcriptional profiles and clear group separation among the control, saline, semaglutide, and tirzepatide groups, underscoring the transcriptomic impact of HFHFD and pharmacological interventions (Figure 4C). Volcano plots and hierarchical clustering heatmaps illustrated distinct DEG distributions and gene expression profiles among the four groups, suggesting condition-specific transcriptional signatures. As shown in Figures 4D,E, the saline group exhibited significant upregulation of several genes, including *Pon1*, *Cyp1a1*, *Il1b*, *Egr2*, *Ryr2*, *Tfrc*, and *Ptger4*, compared with the control group. These genes are potentially implicated in HFHFD-induced pathophysiological processes, such as inflammation, oxidative stress, and metabolic disturbances. Conversely, a set of genes—including *Hsd11b1*, *Camk2b*, *Aacs*, and *Atp1a3*—were significantly downregulated, suggesting reduced activity or suppression of their associated biological functions. Relative to saline treatment, semaglutide administration led to increased expression of *Atp1a3*, *Cyp1a1*, and *Hsd11b1*, whereas *Zbtb16* and *Ryr2* were suppressed. In the tirzepatide group, *Atp1a3*, *Cyp1a1*, *Hsd11b1*, and *Tfrc* were upregulated, whereas *Il1b*, *Ptger4*, and *Tiam1* were downregulated compared with the saline group.

### 3.6 GO and KEGG pathway enrichment analyses

**Figure 5:**
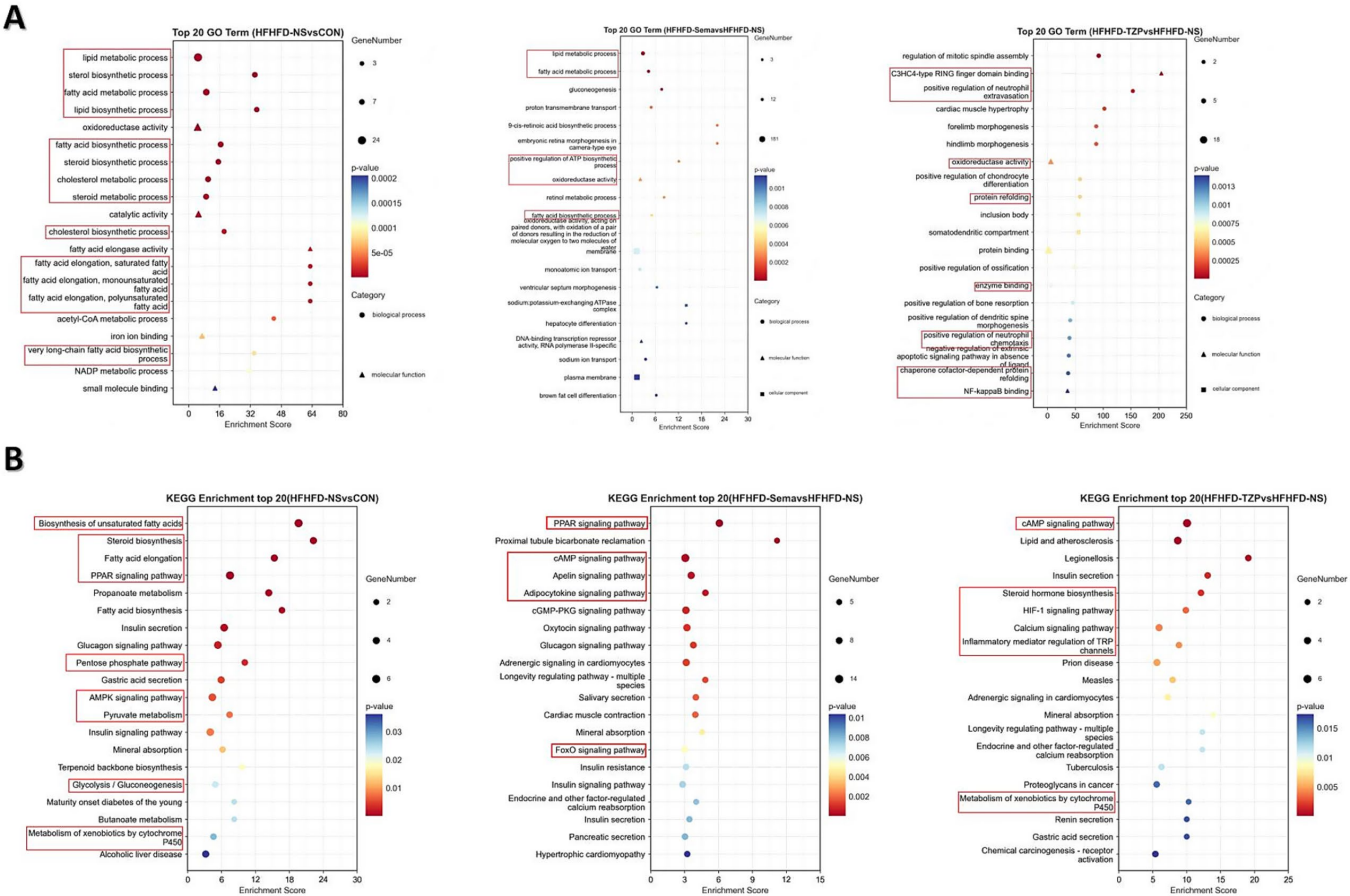
Functional enrichment analysis of DEGs in GO categories and KEGG pathways. **(A)** GO enrichment analysis of DEGs in the categories of biological process (circles), molecular function (triangles), and cellular component (squares). **(B)** KEGG pathway enrichment analysis of DEGs. The x-axis represents the Rich Factor (ratio of observed DEGs to total annotated genes); dot size corresponds to the number of DEGs enriched in each term or pathway; dot color indicates the significance level (adjusted *p*-value). The top 20 enriched GO terms and KEGG pathways are shown. Statistical significance was determined using the hypergeometric test followed by Benjamini–Hochberg adjustment.

GO enrichment analysis demonstrated that DEGs were significantly overrepresented across biological processes (BP), molecular functions (MF), and cellular components (CC), indicating functional relevance at multiple cellular levels. In BAT following HFHFD intervention, DEGs were predominantly involved in BPs such as lipid metabolism, fatty acid metabolic process, and lipid biosynthesis, which are closely associated with energy homeostasis in BAT. These results suggest a potential metabolic remodeling of BAT under HFHFD conditions, with an emphasis on lipid-handling pathways. In the semaglutide group, DEGs were primarily involved in lipid metabolism, fatty acid oxidation, ATP biosynthesis, and oxidoreductase activity. This enrichment indicates that semaglutide may enhance lipid utilization and energy production in BAT, contributing to its metabolic benefits. In contrast, DEGs in the tirzepatide group were predominantly enriched in positive regulation of neutrophil extravasation, oxidoreductase activity, and NF-κB binding. These findings imply that tirzepatide may exert immunomodulatory and anti-inflammatory effects alongside metabolic regulation, potentially contributing to improved metabolic homeostasis (Figure 5A).

KEGG pathway analysis revealed distinct enrichment profiles of differentially expressed mRNAs across the experimental groups. The saline group demonstrated pronounced enrichment of multiple metabolic and signaling pathways—such as PPAR signaling, fatty acid elongation, steroid biosynthesis, and AMPK signaling—relative to the control group. This enrichment suggests notable changes in energy metabolism, inflammatory signaling, and apoptotic processes following HFHFD exposure. In the semaglutide group, DEGs were primarily enriched in pathways involved in inflammatory responses, metabolic control, and disease-associated signaling, implying that semaglutide may contribute to immune regulation and the maintenance of metabolic balance. Tirzepatide-associated DEGs showed significant enrichment in pathways regulating lipid and energy metabolism, glycolytic processes, and insulin signaling, supporting its potential role in restoring metabolic homeostasis and improving systemic energy regulation (Figure 5B).

### 3.7 PPI network analysis

**Figure 6:**
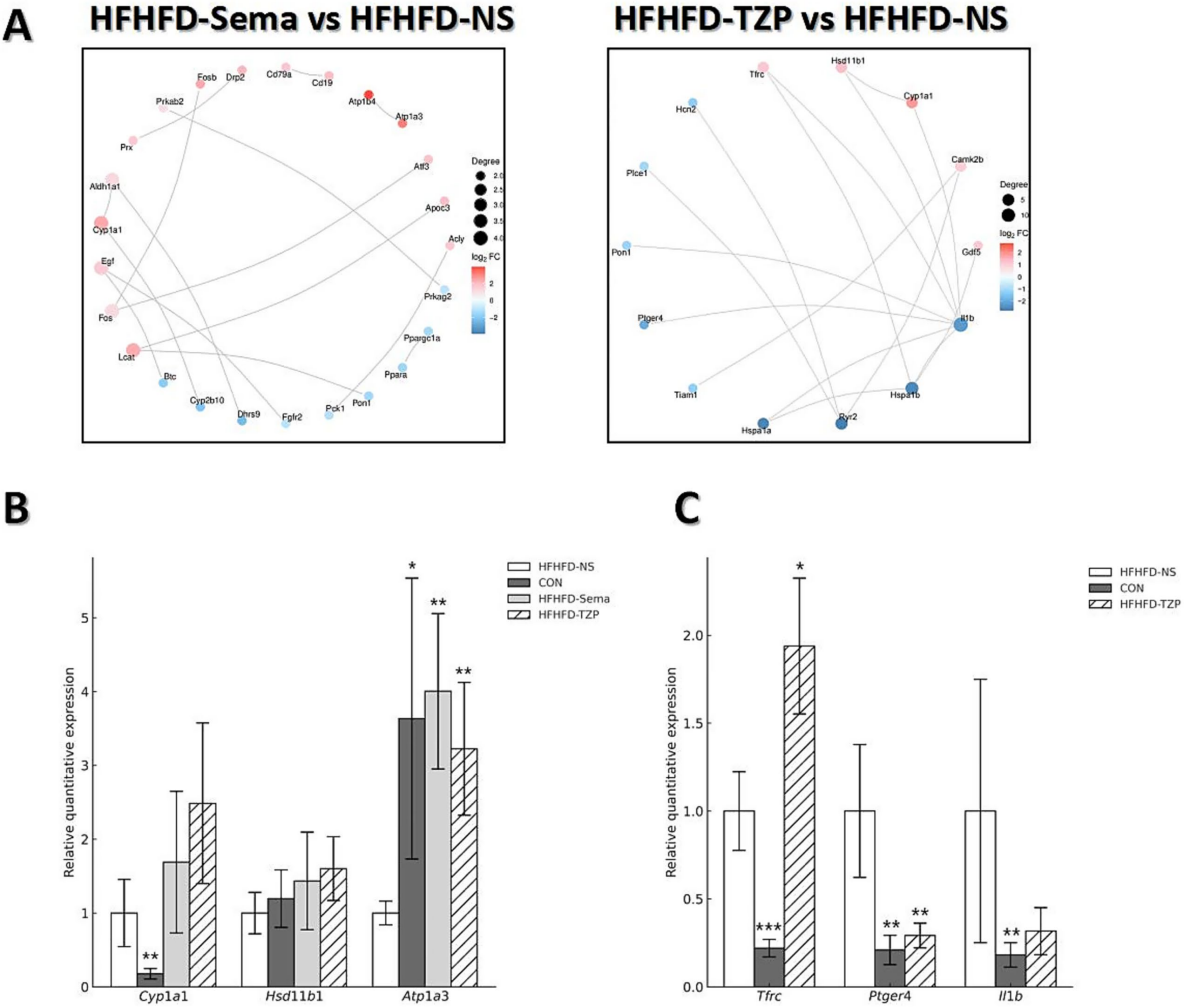
PPI network analysis and validation of gene expression by RT-qPCR. **(A)** PPI networks of the top 30 DEGs in the semaglutide and tirzepatide groups relative to the HFHFD-NS group. Node size represents the number of connections (degree), and edge thickness reflects the confidence of protein–protein interactions. **(B,C)** RT-qPCR validation of selected DEGs (*Cyp1a1*, *Hsd11b1*, *Atp1a3*, *Tfrc*, *Ptger4*, *Il1b*). Statistical analysis was performed using one-way ANOVA followed by Dunnett’s *post-hoc* test, comparing each treatment group with the HFHFD-NS group. Symbols: *p < 0.05, **p < 0.01, ***p < 0.001 vs. HFHFD-NS.

The PPI network constructed via STRING (Figure 6A) revealed functional interactions among DEGs, enabling the prioritization of key regulatory candidates. In the semaglutide group, compared with saline, the PPI network generated from the top 30 DEGs highlighted several highly connected proteins, including *Ins*, *Il6*, *Lep*, and *Pparg*. These nodes demonstrated extensive interactions with other proteins, implying their involvement in modulating metabolic processes such as glycemic control, lipid handling, insulin-related cascades, and inflammatory signaling. In the tirzepatide group, the PPI network revealed upregulation of *Tfrc*, *Hsd11b1*, *Cyp1a1*, *Camk2b*, and *Gdf5*, along with downregulation of *Ptger4* and *Il1b* relative to the saline group. Notably, *Il1b* was identified as a highly connected candidate gene, both in terms of its network degree and its well-documented role in metabolic and inflammatory regulation, suggesting it may play an important role in relevant signaling pathways. These interactions suggest that the network is involved in critical regulatory pathways related to inflammation and metabolic control.

### 3.8 RT-qPCR validation of transcriptomic results

RT-qPCR analysis validated the reliability of the RNA-seq data, demonstrating consistent trends in gene expression between the two methods. As shown in Figure 6B, administration of semaglutide led to the upregulation of *Cyp1a1* (1.69 ± 0.96 vs. 1.00 ± 0.46), *Hsd11b1* (1.43 ± 0.66 vs. 1.00 ± 0.28), and *Atp1a3* (4.00 ± 1.05 vs. 1.00 ± 0.16; *p* < 0.01) compared with the saline group. Similarly, tirzepatide treatment induced an upward trend in the expression of *Cyp1a1* (2.49 ± 1.09), *Hsd11b1* (1.60 ± 0.43), and *Atp1a3* (3.22 ± 0.90; *p* < 0.01) relative to saline. As shown in Figure 6C, tirzepatide also upregulated *Tfrc* (1.94 ± 0.39 vs. 1.00 ± 0.22; *p* < 0.05) while downregulating *Il1b* (0.32 ± 0.13 vs. 1.00 ± 0.75) and *Ptger4* (0.29 ± 0.07 vs. 1.00 ± 0.38; *p* < 0.01) compared with saline. Correlation analysis demonstrated strong agreement between RNA-seq and RT-qPCR results, with R^2^ = 0.8438 for the semaglutide versus saline comparison and R^2^ = 0.9919 for the tirzepatide versus saline comparison. These findings indicate that both semaglutide and tirzepatide improve HFHFD-induced metabolic dysfunction by modulating the expression of genes related to metabolism and energy balance. Moreover, beyond metabolic regulation, tirzepatide appears to exert additional effects on immune-related genes, potentially contributing to further improvement of the obese phenotype—an effect that may partly explain its advantage over semaglutide.

## 4 Discussion

In this study, we observed a pronounced discrepancy in the number of DEGs in BAT between the semaglutide and tirzepatide treatment groups, despite tirzepatide exhibiting better pharmacodynamic efficacy, as evidenced by larger weight loss, improved glycemic control, and enhanced insulin sensitivity. This difference is likely attributable to multiple interrelated factors.

First, the disparity in DEG counts should not be interpreted as a direct reflection of differences in pharmacological potency. The extent of transcriptomic changes does not necessarily correspond to the magnitude of metabolic improvement (30–32). Although both drugs act as GLP-1 receptor agonists, tirzepatide is an unbalanced dual GLP-1/GIP receptor agonist with higher affinity for the GIP receptor. Despite being administered at a lower dose and inducing fewer DEGs, tirzepatide robustly activated key metabolic pathways, suggesting that it may achieve systemic metabolic benefits through the precise modulation of a limited set of pivotal pathways—a “few but critical” regulatory pattern (9). From a systems biology perspective, a smaller DEG set may indicate a more selective transcriptional program with a high signal-to-noise ratio, potentially leading to robust physiological effects with minimal off-target transcriptional perturbations. This aligns with the concept that therapeutic efficacy is not solely determined by the breadth of transcriptomic remodeling, but by the specificity and relevance of the targeted pathways (33). By comparison, semaglutide induced a broader spectrum of transcriptomic changes across multiple metabolic pathways, which may include the activation of compensatory gene networks. In contrast, tirzepatide’s transcriptional response appeared more targeted—particularly toward amino acid metabolism and thermogenesis—rather than involving large-scale transcriptomic remodeling.

Second, the dosing strategy in this study was based on prior high-quality research in similar mouse models, where the two agents were administered at non-equivalent doses (semaglutide 30 nmol/kg vs. tirzepatide 10 nmol/kg) (17–20). Such a non-equivalent dosing scheme may partly underlie the broader transcriptomic response observed in the semaglutide group. Moreover, their pharmacokinetic profiles differ: semaglutide exhibits prolonged GLP-1 receptor engagement at the tested dose, whereas tirzepatide’s dual-receptor activation may elicit maximal metabolic effects at lower exposures, thereby avoiding excessive catabolism in murine models. Although this approach reduced the risk of hypoglycemia and severe weight loss, the several-fold dose difference may represent a potential confounding factor when interpreting BAT transcriptional responses. A higher dose is likely to increase receptor occupancy and prolong effective tissue exposure, thereby amplifying the breadth of transcriptomic changes. We therefore plan to conduct equivalent-exposure and equivalent-efficacy validation experiments to disentangle the contributions of dose disparity and sampling timing, and to clarify whether the observed DEG differences are primarily driven by exposure levels or pharmacodynamic intensity.

Third, DEG detection is influenced by sample size, within-group variability, between-group differences, and the statistical thresholds applied. Higher biological variability within the tirzepatide group could reduce the number of genes meeting statistical significance, even in the presence of true biological effects. PCA indicated partial overlap between the tirzepatide and saline groups, suggesting modest group separation and potentially reduced detection sensitivity. Furthermore, the statistical thresholds applied in this study were relatively stringent (FDR < 0.05 and |log₂FC| > 1); relaxing these cutoffs would increase the number of DEGs detected in the tirzepatide group. Finally, although euthanasia timing, tissue collection time, and ambient temperature were standardized to minimize experimental variability, subtle differences in sampling location or other technical factors cannot be entirely excluded as contributors to the observed results. In summary, the pronounced disparity in DEG counts between semaglutide and tirzepatide is best understood as the outcome of a multifactorial interplay involving (i) distinct pharmacological mechanisms and receptor selectivity, (ii) non-equivalent dosing and consequent differences in transcriptomic breadth, and (iii) biological as well as technical variability, rather than a straightforward difference in therapeutic potency.

Previous studies have demonstrated that both semaglutide and tirzepatide improve insulin sensitivity and glucose metabolism in diet-induced obese (DIO) mouse models (34, 35). However, the specific molecular targets of these agents in BAT have not been fully elucidated, and no prior study has directly compared their transcriptomic effects in this tissue. In this work, we successfully established a mouse model of metabolic disorder using an HFHFD, which induced obesity and impaired glucose tolerance due to chronic energy surplus. Consistent with previous studies, our findings confirm that both semaglutide and tirzepatide improved the metabolic phenotype of HFHFD-fed mice, as evidenced by reduced body weight, improved lipid profiles, and enhanced insulin sensitivity. Importantly, this study represents the first comparative transcriptomic analysis of semaglutide and tirzepatide in BAT, identifying key gene targets through RNA sequencing and RT-qPCR validation, and providing novel insights into their potential roles in mediating energy metabolism and insulin sensitivity.

Transcriptomic analysis revealed that both semaglutide and tirzepatide upregulated the expression of *Cyp1a1*, *Hsd11b1*, and *Atp1a3*, suggesting that these genes may contribute to the observed metabolic improvements.

*Cyp1a1* encodes a cytochrome P450 monooxygenase, a key enzyme involved in the oxidative metabolism of xenobiotics and endogenous substrates. This enzyme is essential for metabolizing both endogenous substances and xenobiotics, and is also involved in inflammation and cancer development (36, 37). Existing studies have demonstrated that dihydroxy octadecenoic acids (DiHOMEs)—metabolites of linoleic acid (LA) generated by cytochrome P450 enzymes—can modulate the thermogenic responses of brown adipocytes to cold exposure. These lipid mediators are increasingly recognized for their roles in energy metabolism, BAT activation, inflammation regulation, and nociception, with broad implications in both physiological and pathological settings (38, 39). Epoxy fatty acids derived from eicosapentaenoic acid (EPA) and docosahexaenoic acid (DHA)—including 17,18-epoxyeicosatetraenoic acid (17,18-EpETE) and 19,20-epoxydocosapentaenoic acid (19,20-EpDPE)—are synthesized via the cytochrome P450 epoxygenase pathway. These metabolites enhance thermogenesis in brown and beige adipocytes through the upregulation of thermogenic markers, including UCP1 (40). Consistent with these findings, our transcriptomic analysis indicates that semaglutide and tirzepatide may regulate BAT function, at least partially, via modulation of *Cyp1a1* expression. This modulation may influence cytochrome P450 enzymatic activity, thereby facilitating thermogenesis and enhancing energy expenditure in BAT.

*Hsd11b1* and *Hsd11b2* cooperatively regulate intracellular glucocorticoid activity: *Hsd11b1* catalyzes the activation of inactive cortisone to cortisol, whereas *Hsd11b2* inactivates cortisol by converting it to cortisone. Excessive *Hsd11b1* activation elevates local glucocorticoid activity in adipose and bone tissues, thereby promoting visceral fat accumulation, impaired insulin sensitivity, and osteoporosis associated with aging. However, emerging evidence highlights a more nuanced role of *Hsd11b1* in metabolic processes. Evidence from previous studies indicates that the obesity-reducing properties of As demonstrated by Lv et al. Salvianolic acid B may be mediated through its regulation of lncRNA-*Hsd11b1* in BAT, leading to altered expression of key mRNAs involved in inflammatory and metabolic pathways (41). Similarly, the metabolic relevance of *Hsd11b1* has also been supported by studies on herbal medicine. For example, mulberry leaf extract has been shown to alleviate obesity by upregulating *Hsd11b1* expression in WAT, thereby modulating lipid metabolism and inflammatory pathways (42). Moreover, Barclay et al. reported that exposure to low-dose dexamethasone upregulated Hsd11b1 levels within brown adipose tissue and enhanced the transcription of thermogenic genes, including *Ucp*1 and *Pgc1α* (43). These findings align with our transcriptomic results, suggesting that semaglutide and tirzepatide may exert their metabolic benefits, at least in part, by modulating *Hsd11b1* activity in BAT, thereby promoting thermogenesis and improving systemic energy homeostasis.

*Atp1a3* produces the α3 isoform of the Na^+^/K^+^-ATPase, a membrane-associated enzyme essential for sustaining ionic gradients and stabilizing the resting membrane potential. Although *Atp1a3* has been extensively characterized for its predominant distribution in the neural system and its association with various neurological disorders (44–46), its role in BAT and metabolic regulation remains largely uncharacterized. We hypothesize that *Atp1a3* may influence central membrane potential, thereby modulating appetite and ultimately affecting systemic metabolic homeostasis. However, this hypothesis remains speculative and warrants further mechanistic investigation.

In addition to shared targets, tirzepatide uniquely modulated *Tfrc*, *Il1b*, and *Ptger4*. These findings suggest that tirzepatide may engage additional or distinct molecular pathways compared with semaglutide, potentially contributing to its enhanced metabolic effects.

*Tfrc* encodes the transferrin receptor, a transmembrane glycoprotein involved in cellular iron regulation, which is essential for preserving intracellular iron balance. *Tfrc* expression was significantly upregulated in BAT following tirzepatide administration. Maintaining iron homeostasis is critical for immune competence, red blood cell production, metabolic regulation, and the control of cell growth (47–50). *Tfrc* is predominantly expressed in brown adipocytes and has been implicated in regulating BAT development and thermogenic activation, possibly through iron-mediated metabolic pathways. Loss of *Tfrc* function in mice has been associated with thermogenic defects, metabolic dysregulation, inflammatory responses, and mitochondrial dysfunction (51). Moreover, in obese individuals, *Tfrc* expression has been reported to inversely correlate with BMI and positively correlate with UCP1 expression (52). Our findings suggest that tirzepatide may ameliorate metabolic dysfunction by upregulating *Tfrc* expression, thereby contributing to improvements in obesity, insulin resistance, and inflammation.

*Il1b* and *Ptger4* are two inflammation-associated genes of significant research interest. *Il1b* serves as a key pro-inflammatory mediator in various chronic conditions, particularly in metabolic disorders, including T2DM, atherosclerosis, and non-alcoholic fatty liver disease (53). Previous studies have linked elevated *Il1b* expression with increased adipose inflammatory responses and aggravated insulin resistance. *Il1b* is known to disrupt insulin signaling pathways by activating NF-κB and reducing insulin receptor substrate (IRS) phosphorylation, thereby contributing to systemic insulin resistance (54). Elevated glucose and fatty acid levels in pancreatic tissue can trigger NLRP3 inflammasome, resulting in IL-1*β* release and subsequent β-cell dysfunction and apoptosis, which ultimately contribute to the pathogenesis of T2DM (55). *Il1b* is a well-established negative regulator of insulin signaling, primarily exerting its effects by downregulating the IRS1/PI3K/Akt axis. Accordingly, pharmacological suppression of *Il1b* is considered a potential strategy for enhancing insulin sensitivity and facilitating the management of T2DM (56–58). In this context, our results indicate that tirzepatide may restore insulin signaling and mitigate insulin resistance, at least in part, through the suppression of *Il1b* expression.

As a receptor for prostaglandin E2 (PGE2), *Ptger4* mediates downstream Gs protein–coupled signaling cascades that are central to the regulation of immune and inflammatory processes. PGE2 signaling through EP2 and EP4 receptors is critically implicated in the pathogenesis of cutaneous inflammation (59, 60). In immune cells, PGE2 signals through *Ptger4* to modulate the functions of T lymphocytes and dendritic cells, promoting Th1 cell differentiation and enhancing IL–23–driven Th17 cell amplification *in vitro*. These findings have led to the proposal of EP4 as a viable target for immunomodulatory intervention (61). In non-obese diabetic (NOD) mice, elevated *Ptger4* expression has been associated with dysregulated innate immune responses, while inhibition of *Ptger4* signaling has been shown to restore interferon-*α*/*β* receptor (IFNAR) signaling, suppress interleukin-1 (IL-1) production, and reduce leukocyte infiltration into pancreatic islets. Thus, modulation of the PGE2–*Ptger4* axis may help reestablish the immune balance between type I interferon (IFN-I) and IL-1 pathways, providing a novel direction for therapeutic intervention in type 1 diabetes mellitus (T1DM) (62). The reduction of *Ptger4* expression in BAT observed after tirzepatide intervention indicates a potential link between its metabolic benefits and immune regulation, particularly by suppressing local inflammation.

KEGG pathway analysis revealed that the differentially expressed mRNAs were involved in several key signaling and metabolic pathways. The KEGG pathway findings demonstrated that transcripts elevated by semaglutide treatment were mainly associated with metabolic pathways involving energy production, amino acid turnover, lipid handling, and xenobiotic metabolism. In addition to metabolic pathways, the upregulated genes were also associated with signaling pathways governing cellular growth, survival, differentiation, and endocrine homeostasis. These changes may reflect enhanced metabolic activity, improved cellular adaptation to energy imbalance, and a potential shift toward immune activation or anti-infective defense mechanisms. The downregulated mRNAs were primarily associated with regulatory pathways such as PPAR, cGMP–PKG, AMPK, and RAAS signaling, potentially reflecting a reduction in overall cellular metabolic burden. Tirzepatide-induced upregulated mRNAs were primarily associated with pathways involved in metabolic regulation, signal transduction, and endocrine function, suggesting enhanced regulation of glucose homeostasis, calcium-phosphate metabolism, and anti-inflammatory processes. In comparison, the downregulated mRNAs in the tirzepatide group were predominantly enriched in signaling and cellular regulatory pathways, such as “inflammatory mediator regulation of TRP channels, “potentially reflecting a suppression of inflammatory activity.

Our findings indicate that semaglutide and tirzepatide may modulate the expression of *Cyp1a1* and *Hsd11b1*, potentially influencing the “steroid hormone biosynthesis” and “metabolism of xenobiotics by cytochrome P450” pathways. These regulatory effects may contribute to the modulation of energy metabolism and maintenance of steroid hormone homeostasis. Notably, the observed upregulation of *Atp1a3* may also be associated with appetite regulation, potentially mediated via the “cAMP signaling pathway.” Additionally, in the tirzepatide group, *Tfrc* was involved in the “TGF-*β* signaling pathway,” whereas *Ptger4* and *Il1b* were predominantly mapped to the “inflammatory mediator regulation of TRP channels” pathway. These findings imply that tirzepatide may specifically modulate BAT via inflammation-related pathways, potentially alleviating systemic metabolic abnormalities through integrated immunometabolic regulation (Table 1).

**Table 1:** Summary of DEGs and enriched KEGG pathways.

Group	KEGG	DEGs
HFHFD-Sema vs. HFHFD-NS and HFHFD-TZP vs. HFHFD-NS	Steroid hormone biosynthesis	*Cyp1a1*↑; H*sd11b1*↑
	Metabolism of xenobiotics by cytochrome P450	*Cyp1a1*↑; H*sd11b1*↑
	cAMP signaling pathway	*Atp1a3*↑
HFHFD-TZP vs. HFHFD-NS	TGF-beta signaling pathway	*Tfrc*↑
	Inflammatory mediator regulation of TRP channels	*Il1b*↓; P*tger4*↓

While the study offers meaningful contributions, its limitations must be acknowledged. The relatively small number of samples may weaken the robustness of statistical inference and limit the generalizability of the results. It may also contribute to increased inter-sample variability, partly explaining the lower number of DEGs identified in the tirzepatide group compared to the semaglutide group. This study also focused exclusively on BAT; future investigations should incorporate multi-tissue transcriptomic analyses to capture systemic regulatory effects. Additionally, as this work was conducted in a mouse model, caution should be exercised when extrapolating the findings to humans, since notable interspecies differences in anatomical distribution, thermogenic activity, and pharmacological responsiveness of BAT may affect translational relevance despite functional similarities. Finally, although several candidate genes were identified, their precise mechanistic roles remain to be elucidated. Further functional studies, such as gene knockout or silencing, are warranted to confirm their relevance.

Notably, the transcriptional alterations observed in BAT carry important translational implications. Human BAT activity has been linked to improved glucose homeostasis, enhanced insulin sensitivity, and reduced cardiometabolic risk. Our findings suggest that GLP-1 receptor agonists and dual GIP/GLP-1 receptor agonists may differentially regulate BAT function, potentially providing a molecular explanation for their distinct clinical effects. These insights underscore the possibility that BAT could serve not only as a therapeutic target of incretin-based interventions but also as a biomarker to inform individualized treatment strategies for obesity and type 2 diabetes. Future clinical studies integrating BAT activity imaging or molecular assessments will be essential to validate the applicability of these preclinical findings to human outcomes.

## 5 Conclusion

This research provides further evidence for the therapeutic efficacy of semaglutide and tirzepatide in mitigating metabolic dysregulation in a diet-induced obesity mouse model. *Cyp1a1*, *Hsd11b1*, and *Atp1a3* may act as common targets of semaglutide and tirzepatide, contributing to improvements in insulin responsiveness and systemic metabolic regulation. Conversely, *Tfrc*, *Ptger4*, and *Il1b* appear uniquely regulated by tirzepatide, highlighting its stronger potential in mediating anti-inflammatory responses and ameliorating metabolic dysfunction. Collectively, these results highlight BAT as a potential target for GLP-1 and GIP/GLP-1 receptor agonists and offer a mechanistic framework for future research into their roles in metabolic homeostasis.

## Data Availability

The original contributions presented in the study are publicly available. This data can be found here: NCBI SRA, accession PRJNA1311175.

## Glossary

17,18-EpETE: 17,18-epoxyeicosatetraenoic acid
19,20-EpDPE: 19,20-epoxydocosapentaenoic acid
ANOVA: analysis of variance
AUC: area under the curve
BP: biological processes
BMI: body mass index
BCAA: branched-chain amino acid
BAT: brown adipose tissue
CDAHFD: choline-deficient L-amino acid-defined high-fat diet
CC: cellular components
CON: control group
DEGs: differentially expressed genes
DIO: diet-induced obese
DiHOMEs: dihydroxy octadecenoic acids
DHA: docosahexaenoic acid
EPA: eicosapentaenoic acid
GO: gene ontology
GLP-1: glucagon-like peptide-1
GLP-1Rs: GLP-1 receptors
GIP: glucose-dependent insulinotropic peptide
GIPRs: GIP receptors
H&E: hematoxylin and eosin
HDL-C: high-density lipoprotein cholesterol
HFD: high-fat diet
HFHFD: high-fat, high-fructose diet
IRS: insulin receptor substrate
IL-1: interleukin-1
IPGTT: intraperitoneal glucose tolerance test
KEGG: Kyoto Encyclopedia of Genes and Genomes
LA: linoleic acid
LDL-C: low-density lipoprotein cholesterol
MTP: microsomal triglyceride transfer protein
MF: molecular functions
NOD: non-obese diabetic
NS: normal saline
ORO: Oil Red O
PCA: principal component analysis
PGE2: prostaglandin E2
PPI: protein–protein interaction
RT-qPCR: reverse transcription quantitative polymerase chain reaction
Sema: semaglutide
SD: standard deviation
TZP: tirzepatide
TC: total cholesterol
TG: triglyceride
T1DM: type 1 diabetes mellitus
T2DM: type 2 diabetes mellitus
IFN-I: type I interferon
UCP1: uncoupling protein 1
VLDL: very low-density lipoprotein
WAT: white adipose tissue
WHO: World Health Organization
