# Supplementary material for: Distinct effects of semaglutide and tirzepatide on metabolic and inflammatory gene expression in brown adipose tissue of mice fed a high-fat, high-fructose diet
Source: Front Nutr. 2025 Sep 11;12:1659233. doi: 10.3389/fnut.2025.1659233 (PMC12460090; doi:10.3389/fnut.2025.1659233)
Supplement: Supplementary file 1 [file Table_1.docx]

**Supplementary TableS1** Primer sequences used for RT-qPCR analysis.

| Gene | Forward primer | Reverse primer |
| --- | --- | --- |
| GAPDH | GCAAGGACACTGAGCAAGA | GGATGGAAATTGTGAGGGAG |
| *Cyp1a1* | GTTTATGACACTGCATGTGG | GAGAAACATGGACATGCAAG |
| *Hsd11b1* | CGGAGCAATTTATTGTCAAGG | GTGGATGTCGTCATGGAAGA |
| *Atp1a3* | CAATCTGTACCTGGGCATAG | GACCATGTTCTTGAAGGACT |
| *Tfrc* | CAGAGCTTGAAGATCGTTAGTA | CCAAATGCTCAATGACCACA |
| *Il1b* | AATCTCCAGGGGACTCCTTA | TCTCTTTGAACAGAATGTGCC |
| *Ptger4* | TCAGGATTGCTTCTGTGAAC | CACTTGATCTTCTCTATGGCT |
